# The role of review structure in perceived helpfulness

**DOI:** 10.1038/s41598-026-41169-z

**Published:** 2026-03-15

**Authors:** Yingyue Luna Luan, Yeun Joon Kim

**Affiliations:** 1 UQ Business School, The University of Queensland, Brisbane, QLD 4072 Australia; 2 Judge Business School, University of Cambridge, Cambridge, CB2 1AG UK; 3 Institute of Metabolic Science, School of Clinical Medicine, University of Cambridge, Cambridge, CB2 0QQ UK

**Keywords:** Feedback, Online review, Feedback structure, Review structure, Human behaviour, Psychology

## Abstract

Online product reviews are a pervasive form of public feedback, yet research on their perceived helpfulness has focused almost exclusively on overall sentiment. Drawing on feedback literature, we propose that review structure—the sequencing of positive and negative content across the evaluative message—shapes helpfulness above and beyond aggregate valence. We conducted an exploratory study by analyzing 195,675 Amazon reviews using growth curve modeling to capture each review’s opening tone and valence trajectory. Results show that the most effective structures depend on product ratings. For highly rated products, reviews that grow increasingly positive are most helpful, while those that turn negative are least. For average-rated products, progressively negative trajectories enhance helpfulness, whereas reviews that start negative and grow positive are least effective. For low-rated products, reviews are judged most helpful when they open constructively before introducing criticism. These findings advance theories of online reviews and feedback by showing that how evaluative information is organized matters as much as what is said.

**Supplementary Information:**

The online version contains supplementary material available at 10.1038/s41598-026-41169-z.

## Introduction

Online product reviews are a pervasive form of public feedback that shapes consumer decisions and firm outcomes. A substantial body of research examines when reviews are judged helpful, highlighting factors such as review length and extremity, overall sentiment (valence) and sentiment intensity, readability and specificity, reviewer reputation and verification, as well as contextual factors including average rating levels, dispersion, review volume, and recency^1–8^. This work explains much of what is said in reviews, who wrote them, and where and when they are seen. We build on this foundation by asking a different question: beyond the content itself, does the way reviewers present it—i.e., the structure, or order, of positive and negative content within a single review—affect its perceived helpfulness?

To address this question, we turn to the broader literature on feedback, viewing online reviews as a form of public, crowd-directed feedback. They are evaluative messages directed at a performance target (e.g., a product, service, or seller) that assess the extent to which the target falls short of, meets, or exceeds reviewers’ expected standards. This conceptualization aligns with the definition of feedback as evaluative information about the gap between a target (e.g., a product, person, or idea) and an expected standard^9^. While online review research focuses on consumer behavior in digital marketplaces, feedback theory offers well-developed perspectives that better suit our research purpose—i.e., examining how message characteristics influence readers’ interpretation, acceptance, and usefulness, which are mechanisms likely operating in both private, dyadic exchanges and public, general-audience contexts. Thus, the insights from decades of feedback research can help explain why the structure of positive and negative content in a review may affect its perceived helpfulness.

Feedback research, including studies on online reviews, has largely examined the isolated effects of positive feedback (indicating performance meets or exceeds expectations) and negative feedback (indicating performance falls short). In practice, however, feedback messages often contain both positive and negative elements, and the sequence in which these appear can vary. For example, a cost-effective laptop may be praised for its specifications and design while criticized for its short battery life. Some reviewers may start with praise before turning to criticism, whereas others do the reverse. We term this arrangement *feedback structure*, defined as the organization of multiple pieces of evaluative information within a single message about a target. This structural arrangement, we argue, can influence how the message is processed and evaluated.

Although scholarly investigations on feedback structure have been relatively scarce, there are prevalent anecdotal observations in our society that exemplify its significance. One common approach is the “feedback sandwich,” where criticism is sandwiched between praises. The underlying assumption is that framing negative content between positive content makes it more palatable, thereby increasing recipients’ receptiveness to the criticism and their perception of its helpfulness^10^. Another well-known model is the Pendleton model, which advocates for a balanced approach that starts with objective facts, then praises, and concludes with criticism^11^. Both approaches propose a three-part structure with a beginning, middle, and ending component, aiming to provide a balanced and actionable message.

Building on these approaches, our research adopts the three-part format as a general framework for feedback structure and explores a broad range of possible structural patterns of feedback. Specifically, we classify structures according to their opening tone (positive, neutral, or negative) and valence trajectory over the remainder of the message (increasing, decreasing, or steady), yielding nine possible structures. This design enables us to assess the prevalence of different structures and their perceived helpfulness. In addition, we suggest that the optimal structure may depend on the performance level of the target. Because feedback is typically aimed at evaluating and improving performance^9^, understanding how the performance level interacts with feedback structure in predicting perceived helpfulness of the feedback is critical. For example, low-performing targets might benefit from early positive framing to build the perception of feedback helpfulness, while high-performing targets may gain more from direct, improvement-oriented critique.

We conduct an exploratory study to examine these ideas in the context of public feedback, i.e., online reviews, where feedback structure is operationalized as the valence trajectory across the beginning, middle, and end of the text. Although the abovementioned models of feedback structure, such as the “feedback sandwich” and the Pendleton model, were developed in longer, interpersonal settings, the same sequencing mechanisms can apply to short, public reviews, which also unfold through an opening, middle, and ending that shape how readers interpret sentiment shifts. Focusing on within-review structure enables us to distinguish reviews that may be similar in overall content but differ in how their evaluative elements are organized. For example, a review that opens positively and then introduces critique may be interpreted differently from one that opens with critique and ends on a positive note, even if their aggregate sentiment is identical. We then examine how various types of feedback structures shape perceived helpfulness in relation to the demonstrated rating of the product.

To identify which feedback/review structures are most effective, we analyzed a large dataset of online product reviews^12–15^. (We use the terms *feedback structure* and *review structure* interchangeably in this manuscript. In the Introduction and Discussion, we use *feedback structure* to emphasize the study’s theoretical grounding in the feedback literature. In the Methods and Results, we use a *review structure* to align with our empirical context of online product reviews.) The dataset covered 5,487 distinct products and contained 195,675 reviews. Each review included the full text, the rating of the product (i.e., product performance), and a helpfulness score based on reader votes (see Fig. S1 for an example). For each review, we divided the text into a beginning, middle, and end. We then computed sentiment scores for each segment. These segment-level sentiment scores form the basis for our analysis of review structure—the trajectory of sentiment from the start to the end of a review (see Fig. 4 for the calculation model).

To test whether the helpfulness of review structures depends on rating context, we classified reviews according to the rating of the product they evaluated: reviews of highly rated products, reviews of average-rated products, and reviews of low-rated products. We focus on review-level ratings because they directly reflect the evaluative context that readers encounter when judging each review’s credibility and helpfulness. Within each group, we applied growth curve modeling (GCM) to capture two parameters for every review: (1) the beginning valence, or the sentiment of the opening segment, and (2) the trajectory, operationalized as the review’s random slope, which reflects the rate and direction of change in valence across the review.

GCM offers several advantages over simpler linear models for our research. First, it explicitly models how review valence changes with its position in the review, rather than reducing the analysis to a single overall valence score. Second, its fixed effects allow us to uncover the most prevalent structure across all reviews. Third, it allows for random effects, so that each review can have its own unique trajectory rather than assuming all reviews follow the same pattern. We combined the review-specific random slopes with beginning valence to examine how different structures influence helpfulness. Unlike simpler linear models that would ignore sequencing or require manually coding discrete categories, GCM quantifies review structure continuously and integrates it directly into the prediction of perceived helpfulness.

## Results

We developed nine different review structures based on their beginning tone (positive, neutral, or negative) and valence trajectory over the remainder of the review (increasing, decreasing, or steady). We labelled these structures Type A through Type I (Table 1). For example, Type A review started positive and became even more so, while Type I started negative and became more negative still.

**Table 1:** Types of Review Structures.

Beginning	Change	Explanation	Shape	Type
Positive	Increase	This review begins on a positive note and steadily builds upon that positivity.		A
Positive	Flat	This review initiates on a positive note and maintains its positive tone without any change in its overall positivity.		B
Positive	Decrease	This review starts off positively but then shows a gradual decrease in its level of positivity.		C
Neutral	Increase	This review initially shows a neutral tone and then experiences an augmentation in positivity.		D
Neutral	Flat	This review begins neutrally and maintains the same neutral tone throughout.		E
Neutral	Decrease	This review begins on a neutral note and then gradually becomes more negative.		F
Negative	Increase	This review, originally negative, undergoes an increase in its level of positivity as it progresses.		G
Negative	Flat	This review begins with a negative tone and remains consistently negative throughout.		H
Negative	Decrease	This review begins negatively and becomes even more negative as it continues.		I

In our analyses, we distinguish between prevalent review structures and helpful review structures. Before assessing helpfulness, we first examined the structure estimates to identify the most prevalent review structures within each rating group, defined as those most commonly adopted by reviewers. For example, reviews of low-rated products may often display an increasingly negative tone, whereas reviews of highly rated products tend to become more positive. However, prevalence does not necessarily imply helpfulness; widely adopted structures may still be perceived as less helpful. We therefore analyze which structures are most effective in enhancing perceived helpfulness by examining the relationship between review structure and helpfulness.

**Fig. 1:**
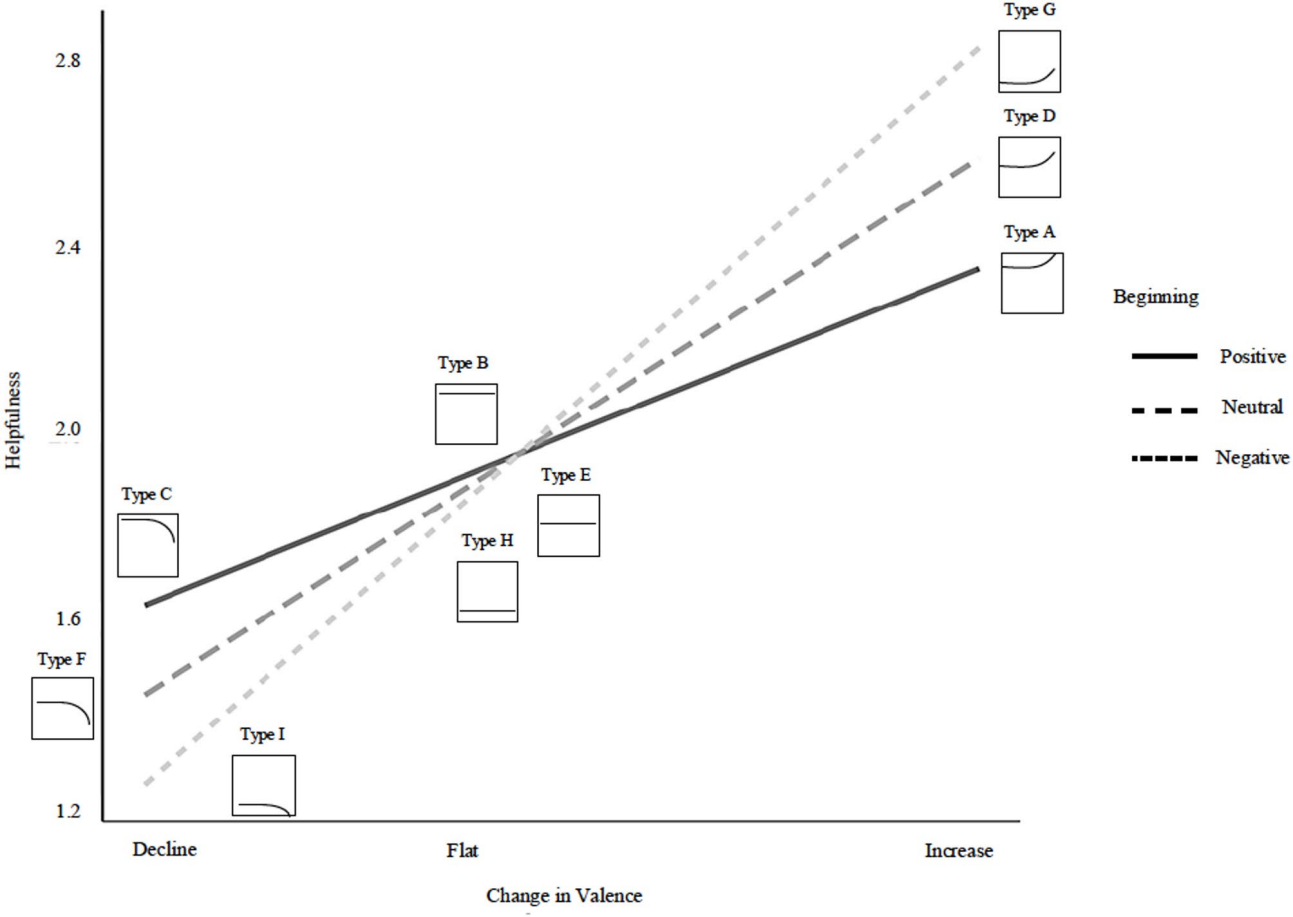
The Interaction of Beginning Valence and Change in Valence Predicting Helpfulness for Reviews of Highly Rated Products.

**Fig. 2:**
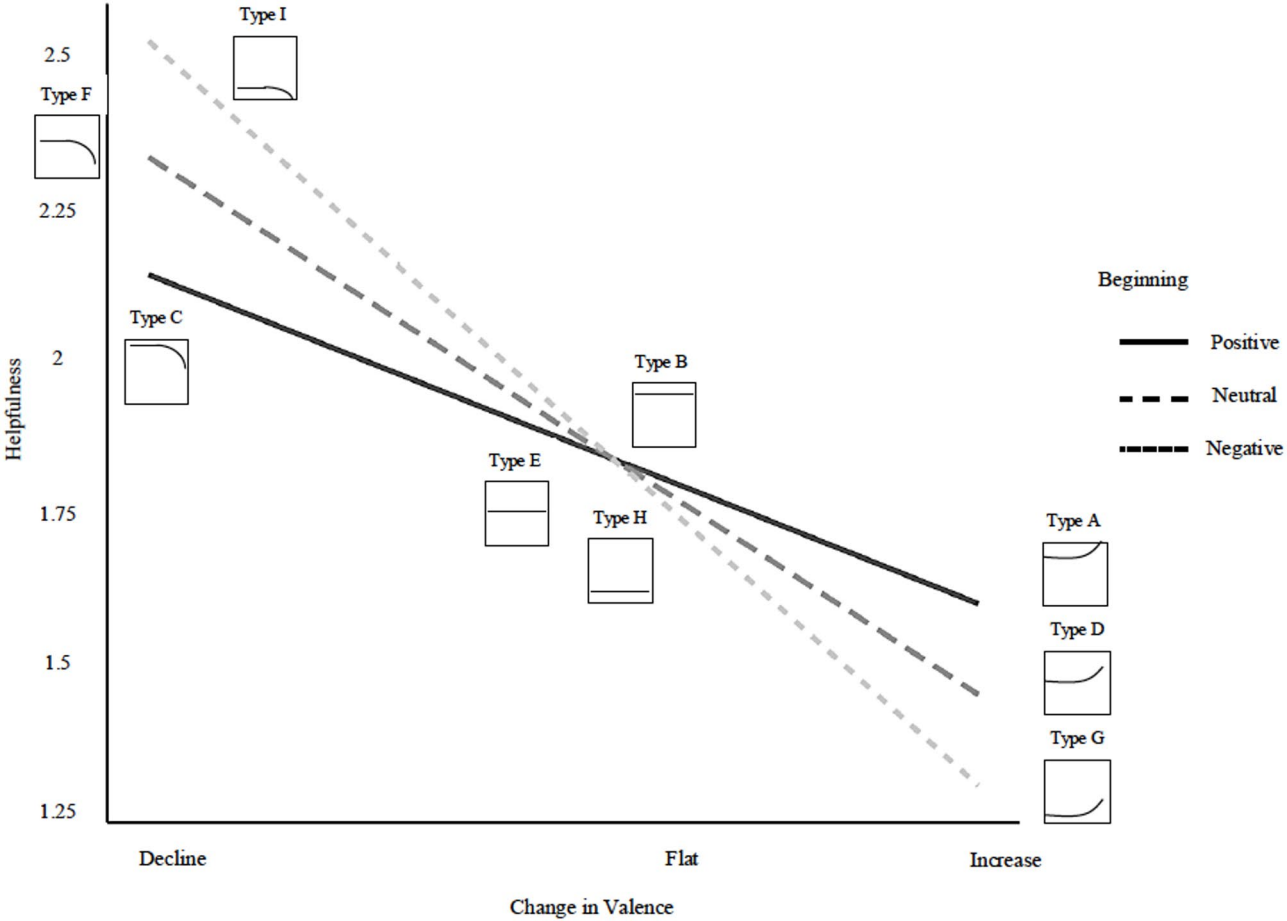
The Interaction of Beginning Valence and Change in Valence Predicting Helpfulness for Reviews of Average-Rated Products.

**Fig. 3:**
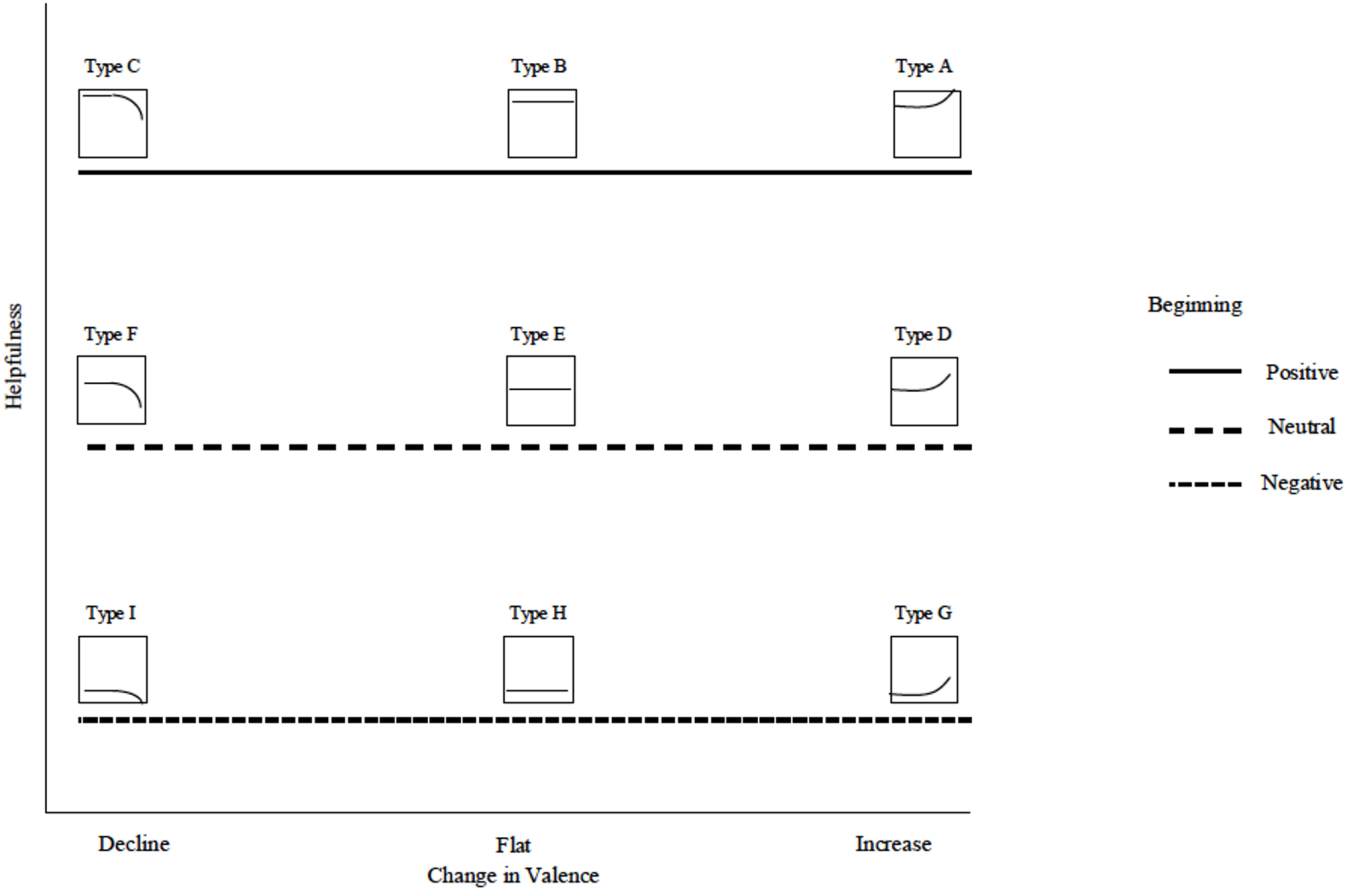
Example of the Interaction of Beginning Valence and Change in Valence Predicting Helpfulness. The x-axis refers to the change in valence. The legend on the right refers to the beginning valence. The y-axis depicts the review helpfulness. According to this plot, Type C (positive beginning and declining change) has the same level of helpfulness as Type B (positive beginning and no change) and Type A (positive beginning and increasing change) but higher helpfulness than Type D, Type E, Type F, Type G, Type H, and Type I.

The benefit of this approach is that it allows us to examine whether the most prevalent structures are also the most helpful. In doing so, we move beyond overall sentiment to connect specific review structures to both their prevalence and their perceived helpfulness. For each rating group (i.e., reviews of highly rated products, reviews of average-rated products, and reviews of low-rated products), we first report the most prevalent review structure and then the most helpful and unhelpful review structure. Full results can be found in Tables S2-S7 in the Supplementary Information. We illustrated all types of review structure and their corresponding impact on helpfulness in Figs. 1 and 2, and a guide to interpreting our figures can be found in Fig. 3.

**Fig. 4:**
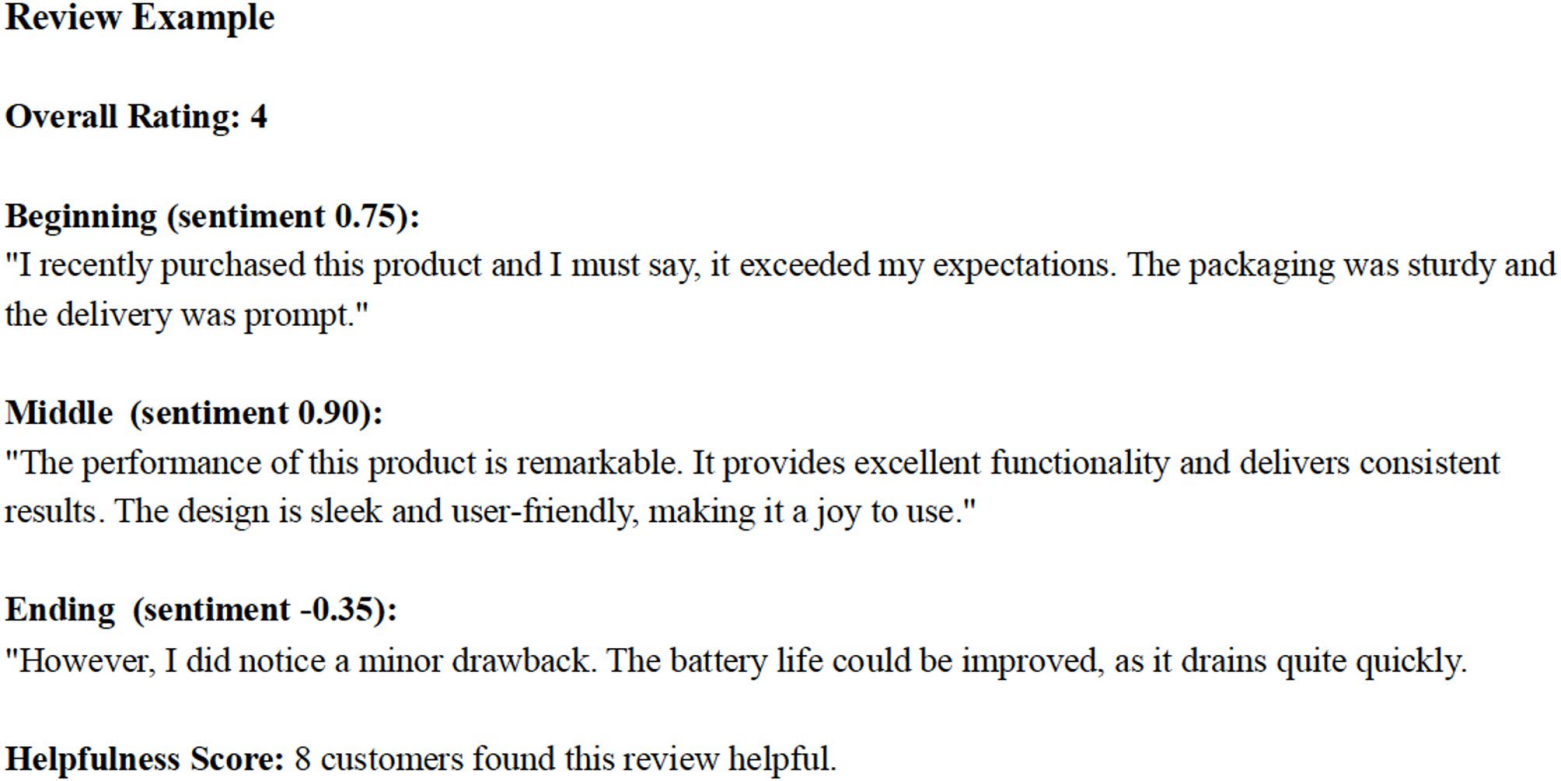
Example Review Structure.

### Reviews of highly rated products

For reviews of highly rated products, the most prevalent structure was Type G, in which review valence consistently increased in positivity (intercept = − 0.23, SE = 0.01, *p* < .001; slope = 0.04, SE = 0.00, *p* < .001; Table S1), indicating that reviews generally become more favorable.

The most helpful structure for reviews of highly rated products was also Type G. That is, reviews that begin negatively but increase in positivity are perceived as most helpful. Such reviews capture attention by initially presenting criticisms, which enhances credibility, before shifting to positive evaluations that frame the product as fundamentally solid. This approach creates the impression of balance and trustworthiness. Importantly, while Type G was the most helpful, its advantage was not statistically different from structures such as Type A or Type D, which also transitioned toward a more positive conclusion from a neutral or positive start (simple slope for Beginning at increasing trajectory: b = 0.00, SE = 0.04, 95% CI [-0.08, 0.08]; Table S3).

In contrast, review structures that progressively decline in valence are linked to a substantial decrease in perceived helpfulness (e.g., Type C, Type F, and Type I). The Type I structure, in which reviews start negatively and decline further, proved the least helpful for reviews of highly rated products. This downward trajectory may foster confusion and discouragement, particularly when the product is generally high quality but the review remains predominantly critical.

### Reviews of average-rated products

The most prevalent structure for reviews of average-rated products was Type G, in which reviews tended to increase in positivity across the review (intercept = − 0.56, SE = 0.02, *p* < .001; slope = 0.09, SE = 0.01, *p* < .001; slope^2^ = 0.05, SE = 0.01, *p* < .001; Table S1). This indicates that reviews for average-rated products often start somewhat negatively but then trend toward more favorable evaluations as they progress.

However, the most helpful structures for reviews of average-rated products were those that decreased in positivity over time, especially Type I, characterized by an initially negative tone that intensifies over the course of the review. Importantly, its effectiveness did not differ statistically from Type F or Type C, all of which shared a strongly negative trajectory regardless of their starting tone (simple slope for Beginning at declining trajectory: b = − 0.05, SE = 0.04, 95% CI [-0.12, 0.02]; Table S5). A commonality among these structures is their escalating negativity, which readers appear to find more informative and diagnostic when evaluating products of middling quality. Simple slopes revealed that increasing trajectories were consistently associated with lower helpfulness, particularly when reviews began negatively (b = -11.14, SE = 1.89, 95% CI [-14.85, -7.43]; Table S5). By contrast, decreasing trajectories were perceived as more useful, likely because they foreground concerns about product limitations rather than closing with reassurance. Compared to reviews of highly rated products, reviews of average-rated products appear more receptive to sustained negativity, perhaps because such reviews highlight areas of improvement for products that have clear weaknesses.

The least helpful structure was Type G, in which reviews began negatively but ended with a more positive tone. Readers may interpret this as non-constructive or even misleading, as it initially raises concerns but then shifts toward positivity in a way that undermines the credibility of the critique. Notably, this finding reveals a striking contrast between the most prevalent and the most effective structures. While Type G was the prevalent structure for reviews of average-rated products, it was simultaneously the least helpful. This misalignment suggests that reviewers of average products often write reviews that begin negatively but then become more positive, but readers prefer sustained criticism (e.g., Type C, Type F, and Type I) when evaluating these mid-tier products. In other words, for average-rated products, what reviewers most often write is not what readers find most useful, highlighting a potential disconnect between reviewer intentions and reader expectations.

### Reviews of low-rated products

The most prevalent structure for reviews of low-rated products was Type G, in which reviews start negatively and then increase in positivity (intercept = − 0.35, SE = 0.04, *p* < .001; slope = 0.20, SE = 0.01, *p* < .001; slope^2^ = 0.12, SE = 0.01, *p* < .001; Table S1). However, this structure was not associated with greater helpfulness. The interaction between beginning valence and trajectory was significant (b = 9.95, SE = 4.82, *p* < .05; Table S6), but simple slopes showed that trajectories were never significantly related to helpfulness, regardless of whether reviews began negatively, neutrally, or positively.

Instead, for the low-rated products, what mattered most was how reviews opened. Reviews that began more positively were rated as more helpful, especially when the review remained steady in tone (Type B; b = 0.21, SE = 0.09, 95% CI [0.03, 0.39]; Table S7) or became slightly more positive (Type A; b = 0.38, SE = 0.13, 95% CI [0.12, 0.63]; Table S7). Beginning on a positive note appears to establish goodwill and foster an open mindset among readers, making them more receptive to the review that follows for low-rated products.

By contrast, the review structures found to be least effective for reviews of low-rated products were those characterized by negative starting points (e.g., Type G, Type H, and Type I). Starting with blunt criticism sets an overly harsh tone from the outset, which can make readers defensive or discouraged, diminishing receptivity to later, more constructive content. It can also render the review redundant, since the product’s low rating already signals dissatisfaction.

Notably, this creates a misalignment in that the most prevalent structure for reviews of low-rated products (Type G) is also among the least effective for perceived helpfulness. In other words, reviewers tend to write reviews that begin critically and attempt to “soften” their stance over time, but readers find such reviews unhelpful. What readers value most are reviews that open on a positive note and then remain consistent or grow more positive.

## Discussion

Our exploratory study examined how the structure of evaluative messages—specifically, the sequencing of positive and negative content across a beginning, middle, and end—shapes perceived helpfulness in online product reviews. Rather than searching for a single “best” approach, we identified how both the most and least effective structures vary by the product’s rating. Three patterns stood out. For highly rated products, reviews that became increasingly positive were most helpful, whereas those that turned progressively more negative were least effective. For average-rated products, the opposite pattern emerged: reviews that became increasingly negative were perceived as most helpful, while reviews that began negatively but shifted toward positivity were least effective. For low-rated products, reviews were most helpful when they began positively and either stayed positive or grew more positive, while those that opened negatively were consistently less effective. We also found that for average- and low-rated products, the structures most frequently used by reviewers were not the ones readers found most helpful, suggesting that commonly adopted patterns are not necessarily effective. This misalignment may stem from differing motivations. Reviewers may write mainly for self-expression or emotional release rather than to maximize helpfulness votes, especially when describing products with moderate or poor performance. Thus, review writing serves not only as a means of information sharing but also as a socially expressive act, which helps explain why prevalent structures do not always align with what readers perceive as most useful.

### Theoretical and practical contributions

Our findings contribute to the literature on online reviews. Prior research has primarily examined reviews in terms of their overall sentiment valence, whether a review is, on average, positive or negative^2,4,6,16^. While this approach has yielded valuable insights, it simplifies the realistic structure of reviews, which often contain a blend of praise and critique. For example, a review might commend a product’s price and convenience while lamenting its durability, yet such complexity is flattened into a single valence score. This aggregation obscures the temporal nature of reviews: what is mentioned first, how the tone shifts, and how the review concludes can all shape how readers interpret and evaluate the message. Our research moves beyond this limitation by showing that the structure of reviews, the sequencing of positive and negative content across the review, matters as much as their overall sentiment. For instance, two reviews may express the same total balance of praise and criticism, yet their helpfulness differs depending on whether they open constructively and then introduce critique or instead start negatively and conclude with reassurance. By demonstrating the importance of review structure, our work broadens the theoretical lens for studying online review helpfulness and highlights a critical yet previously neglected factor—review structure—in shaping perceived helpfulness. Our findings also connect with broader work on primacy-recency effects in communication. Studies show that the position of information—whether it appears first or last—can shape how audiences interpret importance and persuasiveness^17–19^. We extend this research by highlighting the dynamic unfolding of positive and negative content within a message. Our results suggest that contrasting only openings and closings is insufficient for understanding message effectiveness. Instead, the full temporal organization of sentiment—the way positivity and negativity evolve across the beginning, middle, and end—plays a critical role in shaping how messages are interpreted.

Beyond online reviews, our work contributes to the broader literature on feedback by recognizing reviews as a form of public, crowd-directed evaluation. Like workplace or educational feedback, online reviews provide evaluative information about a target, with the goal of helping an audience interpret the target’s performance and make purchase decisions accordingly. Similar to the online review literature, prior research on feedback has largely emphasized the effects of overall valence, whether the feedback is positive or negative^20–22^. However, feedback valence may not influence perceived helpfulness, as feedback valence often is a double-edged sword^23–25^. While positive feedback can enhance motivation and openness, it may also be dismissed as uninformative^23^. Negative feedback, though potentially more diagnostic, can provoke defensiveness and resistance^26^. We offer a different perspective by shifting attention from feedback valence to feedback structure, the organization of positive and negative elements across the beginning, middle, and end of a feedback message.

Our results highlight practical implications for how platforms and companies can redesign review prompts and submission forms to elicit structures that readers find most useful. Because perceived helpfulness depends on both the product’s rating and the sequencing of content (i.e., review structure), firms can move beyond generic “Write your review here” boxes and instead introduce performance-sensitive micro-prompts that gently shape how reviewers structure their feedback. For example, when products are highly rated, platforms can encourage reviewers to begin with any minor issues and then explain what worked especially well overall, which could lead the readers to perceive it as balanced and credible. For average-rated products, the form can instead invite reviewers to “start with what fell short or could be improved, and then add details or examples,” guiding them toward a progressively negative trajectory that readers interpret as more diagnostic for mid-tier products. For low-rated products, review forms can be adjusted to invite reviewers to open constructively by noting “what, if anything, worked for you before sharing your main concerns,” which helps establish goodwill and prevents the review from being dismissed as overly harsh. Such small changes in prompt wording or field order can significantly alter how reviewers structure their narratives, aligning their natural writing flow with the structures that audiences actually value. Importantly, these nudges do not censor or distort authentic consumer voices but instead help reviewers present their thoughts in ways that maximize clarity, credibility, and usefulness. For practitioners, this means that by carefully crafting review prompts and input forms, firms can increase the likelihood that submitted reviews match the structural patterns readers find most informative, thereby improving the perceived quality of the review system as a whole and enhancing customer trust in both the product and the platform.

### Limitations and future research avenues

We acknowledge limitations that suggest future research avenues. First, our analyses focused on reviews long enough to capture structural sequencing, which means our sample likely represents more detailed or engaged reviewers. While shorter reviews may still exhibit similar three-part organization, this remains an empirical question, especially for very brief reviews that may not contain distinct segments. Future research could examine short reviews using alternative methods, such as experimental manipulations, to test whether the same structural dynamics hold under tighter word constraints. Second, early product reviews may shape the tone or content of subsequent ones. To address this possibility, we re-estimated our models using a short-tenure subsample (e.g., reviews posted within a single year, 2010; see Model E in Tables S2, S4, and S6). Results were virtually unchanged, except for low-rated products, which had too few observations for reliable estimation. We also controlled for the timing of review postings (see Model B in Tables S2, S4, and S6), further demonstrating the robustness of our findings to temporal influences. Future experimental replications could test sequencing effects across different posting periods more precisely, helping to disentangle structural dynamics from social influence processes. Lastly, as an exploratory study, our research did not directly test the mechanisms that make particular review structures more or less effective. We encourage future research to investigate the psychological processes underlying these effects. One promising explanation involves expectancy-based mechanisms^27–29^. As our results showed, readers approaching a highly rated product may expect increasingly positive information, so encountering growing criticism may violate that expectation. In contrast, for average-rated products, negative turns often align with readers’ expectations and may therefore appear more diagnostic or credible. When encountering a moderately rated product, readers may typically assume that it has both strengths and weaknesses. As a result, reviews that increasingly highlight shortcomings seem realistic and informative, reinforcing the perception that the reviewer is offering an honest, balanced evaluation rather than an overly positive endorsement. For low-rated products, however, our findings are less easily explained by expectancy-based accounts. Reviews that began positively and maintained a positive tone were judged most helpful, which is counterintuitive since readers would typically expect greater emphasis on the products’ shortcomings. One possibility is that readers already assume poor quality for low-rated products and instead seek positive details to balance their impressions or identify redeeming features. Future research exploring such mechanisms would thus meaningfully advance understanding of how review structure shapes perceived helpfulness.

## Conclusion

This study demonstrates that the helpfulness of feedback depends not only on what is said but also on how it unfolds over time. By modeling the valence trajectory of online reviews, we show that the most effective review structures vary systematically with the product’s rating. Increasingly positive sequences reinforce excellence for highly rated products, progressively negative sequences signal needed change for average-rated products, and positive openings foster receptiveness for low-rated products.

## Methods

### Data

 We drew on four publicly available archival datasets of Amazon product reviews^12,15^. Together, these datasets span reviews across multiple product types, including clothing, musical instruments, food, and electronics. Details about the datasets can be found in Table S8 in the Supplementary Information. Each review contained three key components: (1) the full review text, (2) a rating of the product on a scale from 1 (least favorable) to 5 (most favorable), and (3) a helpfulness score, based on the number of readers who voted the review as helpful.

To capture review structure, we divided each review text into three sequential segments representing the beginning, middle, and end, proportionally by character length into 30%–40%–30% segments (if the split point is at a punctuation, the nearest alphanumeric character will be identified).

 Sentiment valence for each segment was calculated using the Vader sentiment analysis tool, a widely validated method for analyzing online textual data^30–32^. For all analyses, we used the compound score, which integrates positive, negative, and neutral values into a single index normalized between − 1 (most negative) and + 1 (most positive). An illustration of this segmentation and scoring procedure is provided in Fig. 4.

 Because our interest was in the structural trajectory of reviews and their relationship to helpfulness, we applied three exclusion criteria. First, we dropped reviews shorter than 300 characters, as very brief reviews do not provide enough text for meaningful segmentation. Second, we excluded reviews with helpfulness scores beyond ± 2 standard deviations from the mean to reduce the influence of extreme outliers. Third, we excluded products with fewer than 10 reviews to ensure sufficient review data per product and avoid extreme cases where a product has a very limited history. These criteria are consistent with prior research, which shows that longer, more detailed reviews are typically judged as more helpful^33^. The final dataset included 195,675 reviews across 5,487 distinct products. The distribution of the number of reviews per product can be found in Fig. S2. To test whether review structures function differently depending on product rating, we grouped reviews into three groups based on each review’s rating of the product (see Fig. S3 for the distribution of product rating): reviews of highly rated products (score = 5), reviews of average-rated products (score = 2, 3, and 4), and reviews of low-rated products (score = 1). We categorize reviews at the review level because it reflects the evaluative lens through which review readers encounter the text, and prior work shows that review-level star ratings shape how readers interpret and assess the usefulness of review content^1,4^. We provide the descriptive statistics and the distribution of review sentiment by product rating groups in Table S9 and Fig. S4 in the Supplementary Information.

### Analyses

 We analyzed review structures in three steps, conducted separately for reviews of highly rated, reviews of average-rated, and reviews of low-rated products. All analyses were conducted within a multilevel framework that accounts for both review- and product-level variance. First, we applied GCM to capture the trajectory of sentiment across the beginning, middle, and end of each review. GCM is well-suited for this purpose because it simultaneously estimates the average pattern of change in review valence as well as the variation across reviews^34,36^. Fixed effects from these models identify the most prevalent trajectory within each product-rating group (e.g., increasing positivity, declining positivity, or curvilinear patterns). Random effects estimate how much each individual review’s trajectory deviates from that average^34,37^, allowing us to identify whether a given review is steeper, flatter, or more declining than the typical pattern. From these models, we extracted the random slope for each review. This slope represents a review-level indicator of valence trajectory, which, in combination with the beginning valence, captures the unique structure of each review. Finally, we examined how review structure predicts perceived helpfulness. We regressed review helpfulness on the interaction between beginning valence (intercept) and trajectory (random slope). This approach tests whether the helpfulness of reviews depends not only on how they open but also on whether their sentiment becomes more positive, more negative, or remains steady over time. We included review length as a control variable. This analysis allows us to assess the effects of the nine possible review structures on helpfulness across different product-rating contexts.

## Supplementary Information

Below is the link to the electronic supplementary material.

Supplementary Material 1

## Data Availability

All data, code, and materials used in the analysis are available on https://osf.io/23k87/.
