# Supplementary material for: The role of review structure in perceived helpfulness
Source: Sci Rep. 2026 Mar 15;16:13594. doi: 10.1038/s41598-026-41169-z (PMC13121453; doi:10.1038/s41598-026-41169-z)
Supplement: Supplementary file 1 — Supplementary Material 1 [file 41598_2026_41169_MOESM1_ESM.pdf]

The Role of Review Structure in Perceived Helpfulness

Yingyue Luna Luan, Yeun Joon Kim

Supplementary Information

Table S1. Growth Curve Modeling 2

Table S2. The Interaction of Beginning Valence and Change in Valence Predicting Helpfulness for Reviews of Highly Rated Products 3

Table S3. Simple Slopes of the Interaction of Beginning Valence and Change in Valence Predicting Helpfulness for Reviews of Highly Rated Products 4

Table S4. The Interaction of Beginning Valence and Change in Valence Predicting Helpfulness for Reviews of Average-Rated Products 5

Table S5. Simple Slopes of the Interaction of Beginning Valence and Change in Valence Predicting Helpfulness for Reviews of Average-Rated Products 6

Table S6. The Interaction of Beginning Valence and Change in Valence Predicting Helpfulness for Reviews of Low-Rated Products 7

Table S7. Simple Slopes of the Interaction of Beginning Valence and Change in Valence Predicting Helpfulness for Reviews of Low-Rated Products 8

Table S8. Description of Datasets 9

Table S9. Descriptive Statistics 10

Fig. S1. Example Review 11

Fig. S2. Distribution of Number of Reviews per Product 12

Fig. S3. Distribution of Product Rating 13

Fig. S4. Distribution of Sentiment by Product Rating Groups 14

Table S1. Growth Curve Modeling

	Reviews of Highly Rated Products			Reviews of Average-Rated Products			Reviews of Low-Rated Products		
	Review Valence			Review Valence			Review Valence		
Predictors	*Estimates*	*SE*	*p*	*Estimates*	*SE*	*p*	*Estimates*	*SE*	*p*
(Intercept)	-0.23	0.01	**<0.001**	-0.56	0.02	**<0.001**	-0.35	0.04	**<0.001**
Slope	0.04	0.00	**<0.001**	0.09	0.01	**<0.001**	0.20	0.01	**<0.001**
$Slope^2$	-0.00	0.00	0.106	0.05	0.00	**<0.001**	0.12	0.01	**<0.001**
Review Length	0.12	0.00	**<0.001**	0.14	0.00	**<0.001**	0.07	0.01	**<0.001**
**Random Effects**									
σ^2	0.17			0.20			0.27		
$\tau_{00\ Review:Product}$	0.00			0.03			0.03		
$\tau_{00\ Product}$	0.01			0.01			0.01		
$\tau_{11\ Review:Product.Slope}$	0.00			0.00			0.00		
$\tau_{11\ Product.Slope}$	0.00			0.00			0.00		
$\rho_{01\ Review:Product}$	-1.00			1.00			1.00		
$\rho_{01\ Product}$	0.20			0.13			0.45		
N_{Review}	95196			50649			8810		
$N_{Product}$	3342			1637			498		
Observations	285588			151947			26430		

Notes. Slope is coded as beginning = -2, middle = -1, and ending = 0.

Table S2. The Interaction of Beginning Valence and Change in Valence Predicting Helpfulness for Reviews of Highly Rated Products

	Model A			Model B			Model C			Model D			Model E			Model F		
	Helpfulness			Helpfulness			Helpfulness			Helpfulness			Helpfulness			Helpfulness		
Predictors	*Estimates*	*SE*	*p*	*Estimates*	*SE*	*p*	*Estimates*	*SE*	*p*	*Estimates*	*SE*	*p*	*Estimates*	*SE*	*p*	*Estimates*	*SE*	*p*
(Intercept)	-5.40	0.15	**<0.001**	27.88	0.37	**<0.001**	-5.38	0.15	**<0.001**	-1.01	0.03	**<0.001**	-5.77	0.46	**<0.001**	-8.33	0.36	**<0.001**
Beginning	0.08	0.04	0.055	0.09	0.04	**0.039**	0.08	0.05	0.096	0.01	0.01	0.256	0.24	0.19	0.195	0.11	0.09	0.246
Change in Valence	10.72	1.91	**<0.001**	9.07	1.60	**<0.001**	9.43	1.62	**<0.001**	2.28	0.40	**<0.001**	25.50	8.75	**0.004**	4.08	1.84	**0.027**
Review Length	1.14	0.02	**<0.001**	1.06	0.02	**<0.001**	1.14	0.02	**<0.001**	0.26	0.00	**<0.001**	1.16	0.07	**<0.001**	1.59	0.05	**<0.001**
Review Time				-0.00	0.00	**<0.001**												
Beginning × Change in Valence	-8.00	2.25	**<0.001**	-7.32	1.80	**<0.001**	-11.53	1.82	**<0.001**	-1.29	0.47	**0.006**	-14.67	7.78	0.060	-5.49	1.91	**0.004**
**Random Effects**																		
σ^2	11.10			9.62			11.10			0.47			6.92			18.37		
$\tau_{00\ Product}$	1.37			3.98			1.37			0.11			1.10			2.38		
ICC	0.11			0.29			0.11			0.18			0.14			0.11		
$N_{Product}$	3342			3224			3342			3342			329			1601		
Observations	95196			90586			95196			95196			5844			38530		
Marginal R^2 / Conditional R^2	0.027 / 0.134			0.163 / 0.408			0.027 / 0.134			0.031 / 0.208			0.048 / 0.179			0.048 / 0.179		

Notes. Model A: Baseline model reported in the manuscript. Model B: Model controlling for review time (applied only to datasets where review time is available; three datasets). Model C: Model using 33%-34%-33% segmentation. Model D: Model incorporating a log-transformed helpfulness score. Model E: Model with reviews posted in 2010. Model F: Model with a minimum review length of 500 characters.

Table S3. Simple Slopes of the Interaction of Beginning Valence and Change in Valence Predicting Helpfulness for Reviews of Highly Rated Products

	Estimate	*SE*	Lower CI	Upper CI	*z*	*p*
Negative Beginning	14.72	2.67	9.49	19.94		
Neutral Beginning	10.72	1.91	6.97	14.46		
Positive Beginning	6.72	1.66	3.47	9.96		
Decrease Change	0.20	0.06	0.06	0.28		
Flat Change	0.08	0.04	-0.00	0.17		
Increase Change	0.00	0.04	-0.08	0.08		
Difference (Negative Beginning Slope – Neutral Beginning Slope)	4.00	1.13			3.55	0.001
Difference (Negative Beginning Slope – Positive Beginning Slope)	8.00	2.25			3.55	0.001
Difference (Neutral Beginning Slope – Positive Beginning Slope)	4.00	1.13			3.55	0.001
Difference (Decrease Change Slope – Flat Change Slope)	0.08	0.02			3.55	0.001
Difference (Decrease Change Slope – Increase Change Slope)	0.17	0.05			3.55	0.001
Difference (Flat Change Slope – Increase Change Slope)	0.08	0.02			3.55	0.001

Table S4. The Interaction of Beginning Valence and Change in Valence Predicting Helpfulness for Reviews of Average-Rated Products

	Model A			Model B			Model C			Model D			Model E			Model E		
	Helpfulness			Helpfulness			Helpfulness			Helpfulness			Helpfulness			Helpfulness		
Predictors	*Estimates*	*SE*	*p*	*Estimates*	*SE*	*p*	*Estimates*	*SE*	*p*	*Estimates*	*SE*	*p*	*Estimates*	*SE*	*p*	*Estimates*	*SE*	*p*
(Intercept)	-3.04	0.17	**<0.001**	23.05	0.51	**<0.001**	-3.03	0.17	**<0.001**	-0.59	0.04	**<0.001**	-3.67	0.59	**<0.001**	-4.36	0.32	**<0.001**
Beginning	0.04	0.03	0.235	0.02	0.03	0.578	0.04	0.03	0.260	0.02	0.01	**0.006**	0.35	0.11	**0.002**	-0.09	0.05	0.076
Change in Valence	-8.03	1.19	**<0.001**	-9.54	1.21	**<0.001**	-8.48	1.24	**<0.001**	-2.50	0.27	**<0.001**	-9.37	2.58	**<0.001**	-11.00	2.43	**<0.001**
Review Length	0.75	0.03	**<0.001**	0.79	0.02	**<0.001**	0.74	0.03	**<0.001**	0.10	0.01	**<0.001**	0.76	0.09	**<0.001**	0.95	0.05	**<0.001**
Review Time				-0.00	0.00	**<0.001**												
Beginning × Change in Valence	6.21	1.83	**0.001**	4.53	1.85	**0.014**	7.63	1.85	**<0.001**	1.42	0.41	**<0.001**	11.18	4.07	**0.006**	5.05	3.51	0.150
**Random Effects**																		
σ^2	7.96			6.42			7.96			0.39			4.93			9.78		
$\tau_{00\ Product}$	1.61			4.51			1.61			0.11			0.98			2.71		
ICC	0.17			0.41			0.17			0.22			0.17			0.22		
$N_{Product}$	1637			1523			1637			1637			110			917		
Observations	50649			46838			50649			50649			2584			26614		
Marginal R^2 / Conditional R^2	0.017 / 0.183			0.137 / 0.493			0.017 / 0.183			0.022 / 0.241			0.037 / 0.197			0.015/ 0.229		

Notes. Model A: Baseline model reported in the manuscript. Model B: Model controlling for review time (applied only to datasets where review time is available; three datasets). Model C: Model using 33%-34%-33% segmentation. Model D: Model incorporating a log-transformed helpfulness score. Model E: Model with reviews posted in 2010. Model F: Model with a minimum review length of 500 characters.

Table S5. Simple Slopes of the Interaction of Beginning Valence and Change in Valence Predicting Helpfulness for Reviews of Average-Rated Products

	Estimate	*SE*	Lower CI	Upper CI	*z*	*p*
Negative Beginning	-11.14	1.90	-14.85	-7.43		
Neutral Beginning	-8.03	1.19	-10.37	-5.70		
Positive Beginning	-4.93	0.97	-6.84	-3.03		
Decrease Change	-0.05	0.04	-0.12	0.02		
Flat Change	0.04	0.03	-0.03	0.11		
Increase Change	0.13	0.05	0.04	0.23		
Difference (Negative Beginning Slope – Neutral Beginning Slope)	-3.10	0.92			-3.38	0.002
Difference (Negative Beginning Slope – Positive Beginning Slope)	-6.21	1.84			-3.38	0.002
Difference (Neutral Beginning Slope – Positive Beginning Slope)	-3.10	0.82			-3.38	0.002
Difference (Decrease Change Slope – Flat Change Slope)	-0.10	0.03			-3.38	0.002
Difference (Decrease Change Slope – Increase Change Slope)	-0.18	0.05			-3.38	0.002
Difference (Flat Change Slope – Increase Change Slope)	-0.10	0.0			-3.38	0.002

Table S6. The Interaction of Beginning Valence and Change in Valence Predicting Helpfulness for Reviews of Low-Rated Products

	Model A			Model B			Model C			Model D			Model E			Model E		
	Helpfulness			Helpfulness			Helpfulness			Helpfulness			Helpfulness			Helpfulness		
Predictors	*Estimates*	*SE*	*p*	*Estimates*	*SE*	*p*	*Estimates*	*SE*	*p*	*Estimates*	*SE*	*p*	*Estimates*	*SE*	*p*	*Estimates*	*SE*	*p*
(Intercept)	-4.06	0.55	**<0.001**	16.54	1.36	**<0.001**	-4.03	0.55	**<0.001**	-0.24	0.10	**0.023**	11.99	2.00	**<0.001**	-9.20	1.45	**<0.001**
Beginning	0.21	0.09	**0.025**	0.27	0.09	**0.003**	0.16	0.09	0.066	-0.01	0.02	0.534	-0.07	0.32	0.819	0.41	0.20	**0.034**
Change in Valence	-2.45	2.89	0.397	-2.30	2.82	0.414	-3.65	2.44	0.135	-0.08	0.54	0.887	-16.41	4.18	**<0.001**	-11.49	3.78	**0.002**
Review Length	1.14	0.08	**<0.001**	1.00	0.09	**<0.001**	1.13	0.08	**<0.001**	0.21	0.02	**<0.001**	-0.94	0.28	**0.001**	1.96	0.21	**<0.001**
Review Time				-0.00	0.00	**<0.001**												
Beginning × Change in Valence	9.95	4.82	**0.039**	9.77	4.72	**0.038**	11.82	3.99	**0.003**	-0.65	0.90	0.467	-23.66	6.20	**<0.001**	3.78	6.15	0.539
**Random Effects**																		
σ^2	16.39			15.74			16.39			0.56			11.07			32.37		
$\tau_{00\ Product}$	2.03			4.85			2.03			0.12			4.61			3.04		
ICC	0.11			0.24			0.11			0.198			0.29			0.09		
$N_{Product}$	498			494			498			498			29			197		
Observations	8810			8766			8810			8810			363			3090		
Marginal R^2 / Conditional R^2	0.025 / 0.132			0.098 / 0.311			0.025 / 0.132			0.020 / 0.198			0.072 / 0.345			0.040 / 0.123		

Notes. Model A: Baseline model reported in the manuscript. Model B: Model controlling for review time (applied only to datasets where review time is available; three datasets). Model C: Model using 33%-34%-33% segmentation. Model D: Model incorporating a log-transformed helpfulness score. Model E: Model with reviews posted in 2010. Model F: Model with a minimum review length of 500 characters.

Table S7. Simple Slopes of the Interaction of Beginning Valence and Change in Valence Predicting Helpfulness for Reviews of Low-Rated Products

	Estimate	*SE*	Lower CI	Upper CI	*z*	*p*
Negative Beginning	-7.43	4.29	-15.84	0.98		
Neutral Beginning	-2.45	2.89	-8.12	3.22		
Positive Beginning	2.52	3.15	-3.65	8.70		
Decrease Change	0.04	0.12	-0.19	0.27		
Flat Change	0.21	0.09	0.03	0.39		
Increase Change	0.38	0.13	0.12	0.63		
Difference (Negative Beginning Slope – Neutral Beginning Slope)	-4.98	2.41			-2.07	0.097
Difference (Negative Beginning Slope – Positive Beginning Slope)	-9.95	4.82			-2.07	0.097
Difference (Neutral Beginning Slope – Positive Beginning Slope)	-7.98	2.41			-2.07	0.097
Difference (Decrease Change Slope – Flat Change Slope)	-0.17	0.08			-2.07	0.097
Difference (Decrease Change Slope – Increase Change Slope)	-0.34	0.16			-2.07	0.097
Difference (Flat Change Slope – Increase Change Slope)	-0.17	0.08			-2.07	0.097

Table S8. Description of Datasets

Dataset	Product Types	Number of Products	Number of Reviews	Average Review Length (in Characters)	Standard Deviation of Review Length (in Characters)	Average of Sentiment of Beginning	Average of Sentiment of Middle	Average of Sentiment of Ending	Standard Deviation of Sentiment of Beginning	Standard Deviation of Sentiment of Middle	Standard Deviation of Sentiment of Ending
1	Clothing	175	9870	433.42	70.03	0.48	0.39	0.37	0.37	0.41	0.42
2	Musical instruments	73	1221	671.67	508.17	0.35	0.39	0.43	0.43	0.45	0.42
3	Fine food	5016	176238	695.43	512.64	0.40	0.40	0.45	0.47	0.51	0.47
4	Electronics	224	8346	810.79	798.49	0.26	0.20	0.25	0.48	0.50	0.48
Total		5487	195675	686.99	519.33	040	0.19	0.44	0.46	0.50	0.47

Table S9. Descriptive Statistics

**Reviews of Highly Rated Products ($N_{Product}$ = 3342, N_{Review} = 95196)**						
Variable	*M*	*SD*	1	2	3	4
1. Helpfulness	1.84	3.57				
2. Review Length	656.65	487.36	.16**			
3. Beginning Sentiment	0.45	0.44	-.02**	.09**		
4. Middle Sentiment	0.50	0.45	-.03**	.11**	.07**	
5. Ending Sentiment	0.54	0.41	0.00	.15**	.06**	.12**

**Reviews of Average-Rated Products ($N_{Product}$ = 1637, N_{Review} = 50649)**						
Variable	*M*	*SD*	1	2	3	4
1. Helpfulness	1.57	3.19				
2. Review Length	730.76	542.44	.10**			
3. Beginning Sentiment	0.39	0.46	-.02**	.11**		
4. Middle Sentiment	0.34	0.51	-.03**	.14**	.09**	
5. Ending Sentiment	0.39	0.47	-.02**	.16**	.10**	.16**

**Reviews of Low-Rated Products ($N_{Product}$ = 498, N_{Review} = 8810)**						
Variable	*M*	*SD*	1	2	3	4
1. Helpfulness	3.09	4.3				
2. Review Length	705.6	594.53	.17**			
3. Beginning Sentiment	0.16	0.51	.02**	.12**		
4. Middle Sentiment	0.00	0.55	.01*	-.04**	.05**	
5. Ending Sentiment	0.08	0.54	.03**	.07**	.06**	.10**

Notes. *M* and *SD* are used to represent mean and standard deviation, respectively. * indicates $p < .05$. ** indicates $p < .01$.

Fig. S1. Example Review

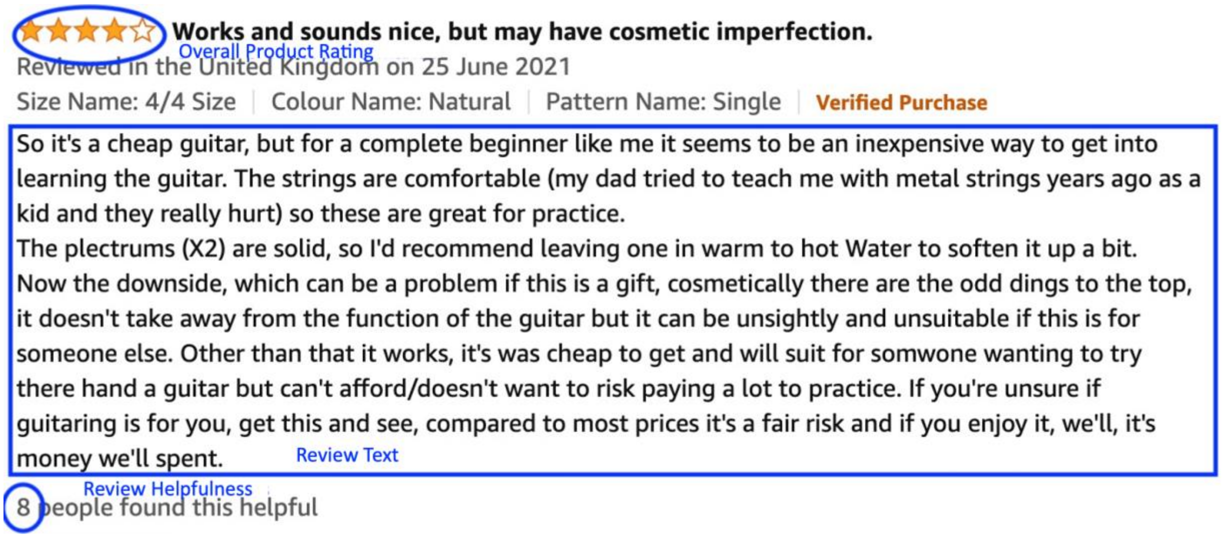

Fig. S2. Distribution of Number of Reviews per Product

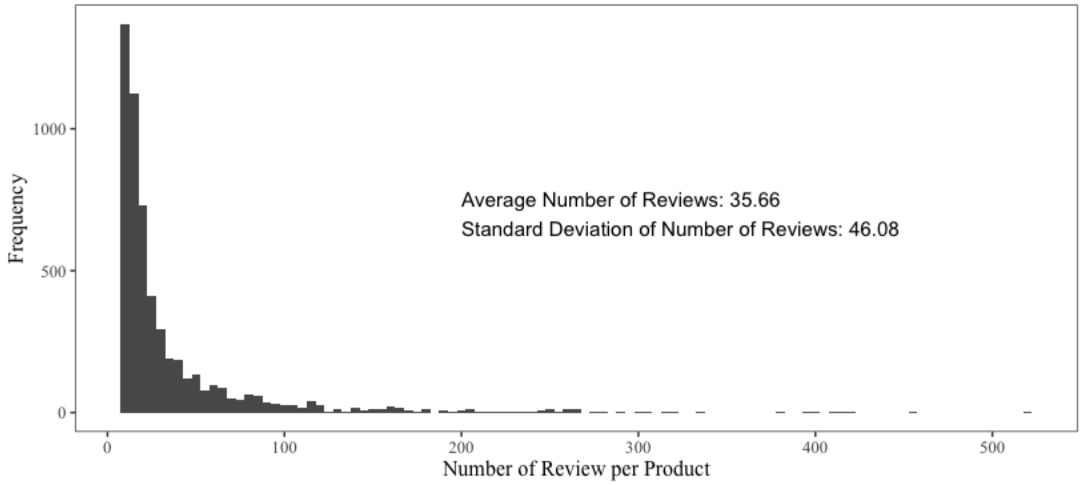

Fig. S3. Distribution of Product Rating

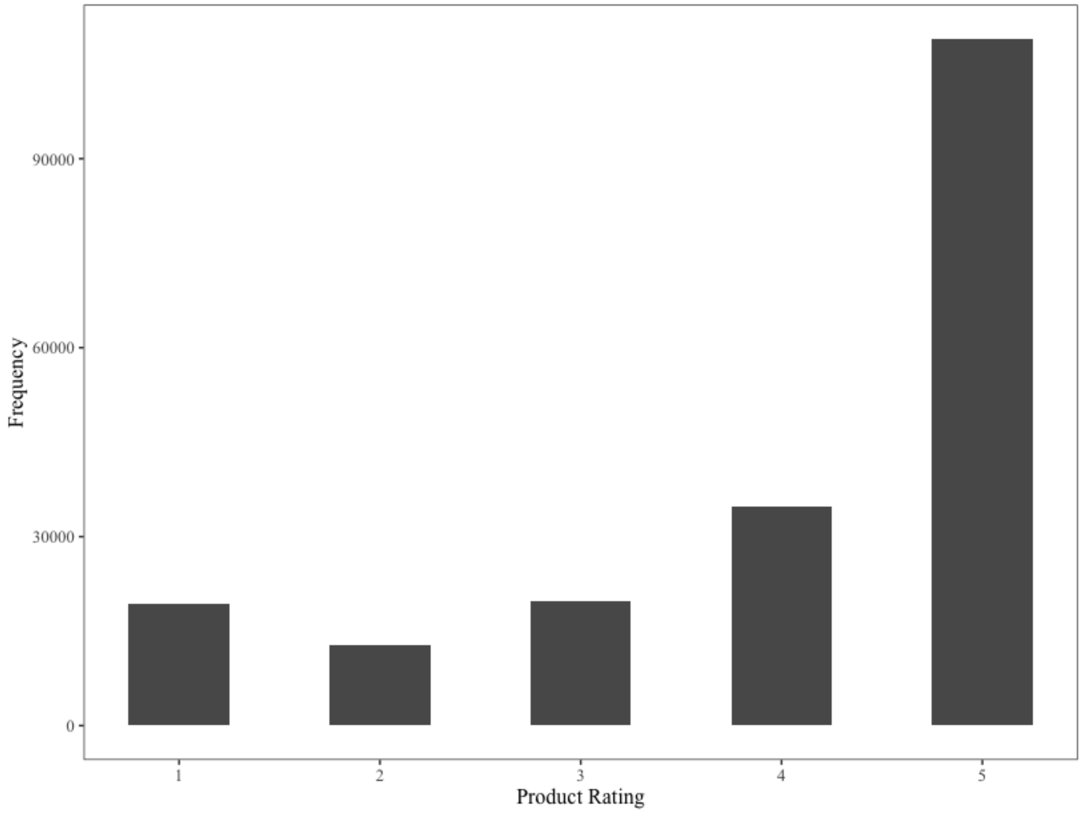

Fig. S4. Distribution of Sentiment by Product Rating Groups

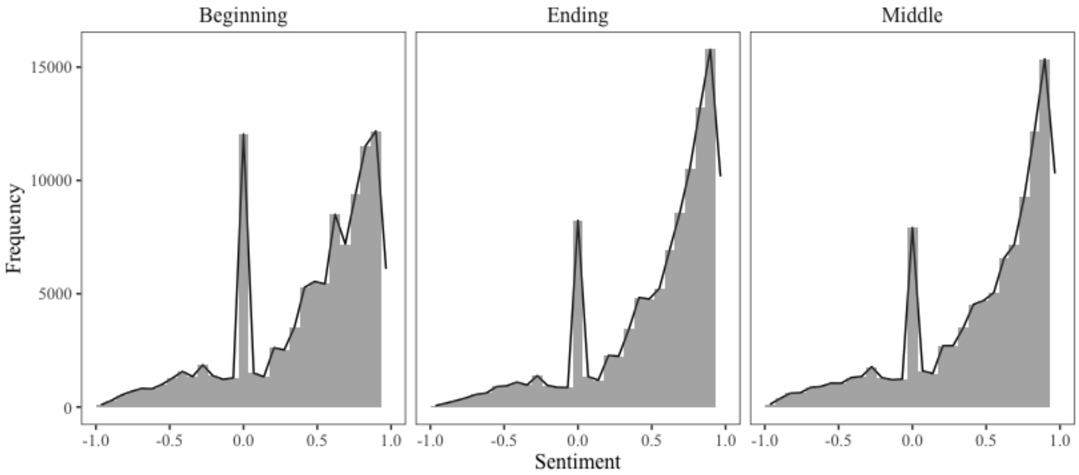

a. Reviews of Highly Rated Products

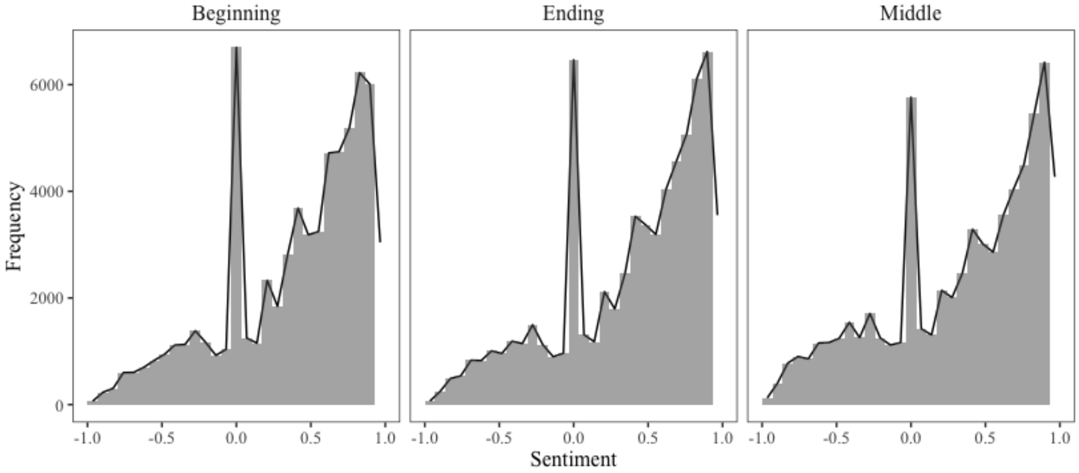

b. Reviews of Average-Rated Products

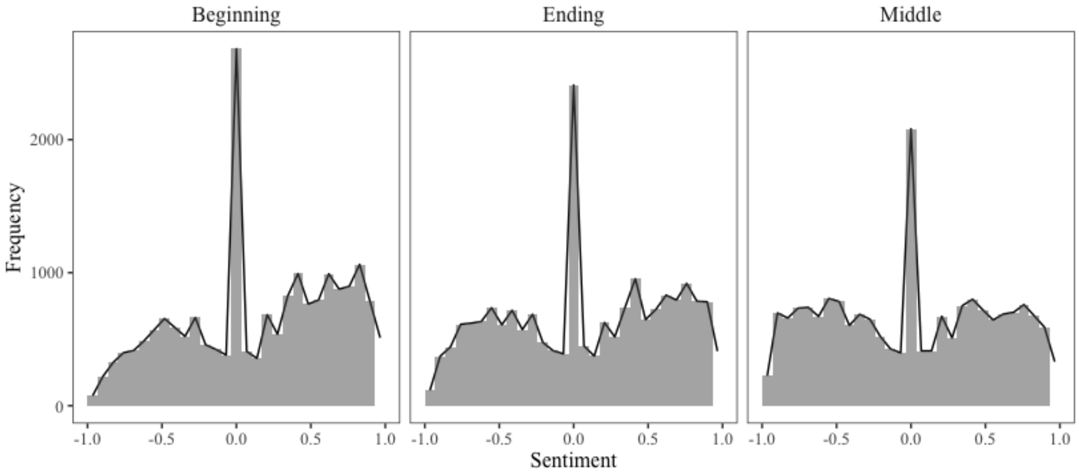

c. Reviews of Low-Rated Products
